# Transient J wave-like ST-segment elevation in intracerebral hemorrhage: a case report

**DOI:** 10.1186/s12872-022-02696-5

**Published:** 2022-06-07

**Authors:** Xing Du, Yongjun Zhang

**Affiliations:** Department of Electrophysiology, Yijishan Hospital of Wannan Medical College, 2 Zheshan Road, Wuhu, China

**Keywords:** Intracerebral hemorrhage, J wave, ST-segment elevation, Myocardial lesion

## Abstract

**Background:**

Certain cerebrovascular events can induce electrocardiography (ECG) abnormalities and cardiac dysfunction. The most frequent patterns reported are nonspecific ST-T change, inverted or broad T wave, prolongation of QT interval as well as ST-segment depression or elevation. Here we present a case of intracerebral hemorrhage (ICH) with transient J wave-like ST-segment elevation accompanied by myocardial lesion.

**Case presentation:**

A 58-year-old woman was admitted to our hospital and diagnosed with right basal ganglia region cerebral hemorrhage. The ECG recorded on the second hospital day showed transient J wave-like ST-segment elevation accompanied by increased myocardial troponin I and myocardial enzyme.

**Conclusions:**

The J wave-like ST-segment elevation may be not a specific ECG signs for primary ischemic heart diseases as it also could be found in ICH patients. We believe that the follow-up ECGs can be used in conjunction with repeated myocardial enzyme analysis and echocardiography to differentiate ICH-ralated J wave-like ST-segment elevation from acute myocardial infarction (AMI), thus avoiding unnecessary cardiac catheterization.

## Background

Certain cerebrovascular events, such as intracerebral hemorrhage (ICH), subarachnoid hemorrhage and intracranial hypertension, can be the cause of electrocardiography (ECG) abnormalities and cardiac dysfunction [1]. The most frequent ECG alterations reported in ICH are nonspecific ST-T change, inverted or broad T wave, prolongation of QT interval as well as ST segment depression or elevation [2]. The observation on electrocardiography of the J wave in hypothermic subjects was first described by Osborn [3]. Since then, a host of J wave–related clinical phenomena, including vasospastic angina, Brugada syndrome, and intracranial hypertension, have been reported [4]. Herein, we present a case of ICH with transient J wave-like ST-segment elevation accompanied by myocardial lesion.

## Case report

**Fig. 1:**
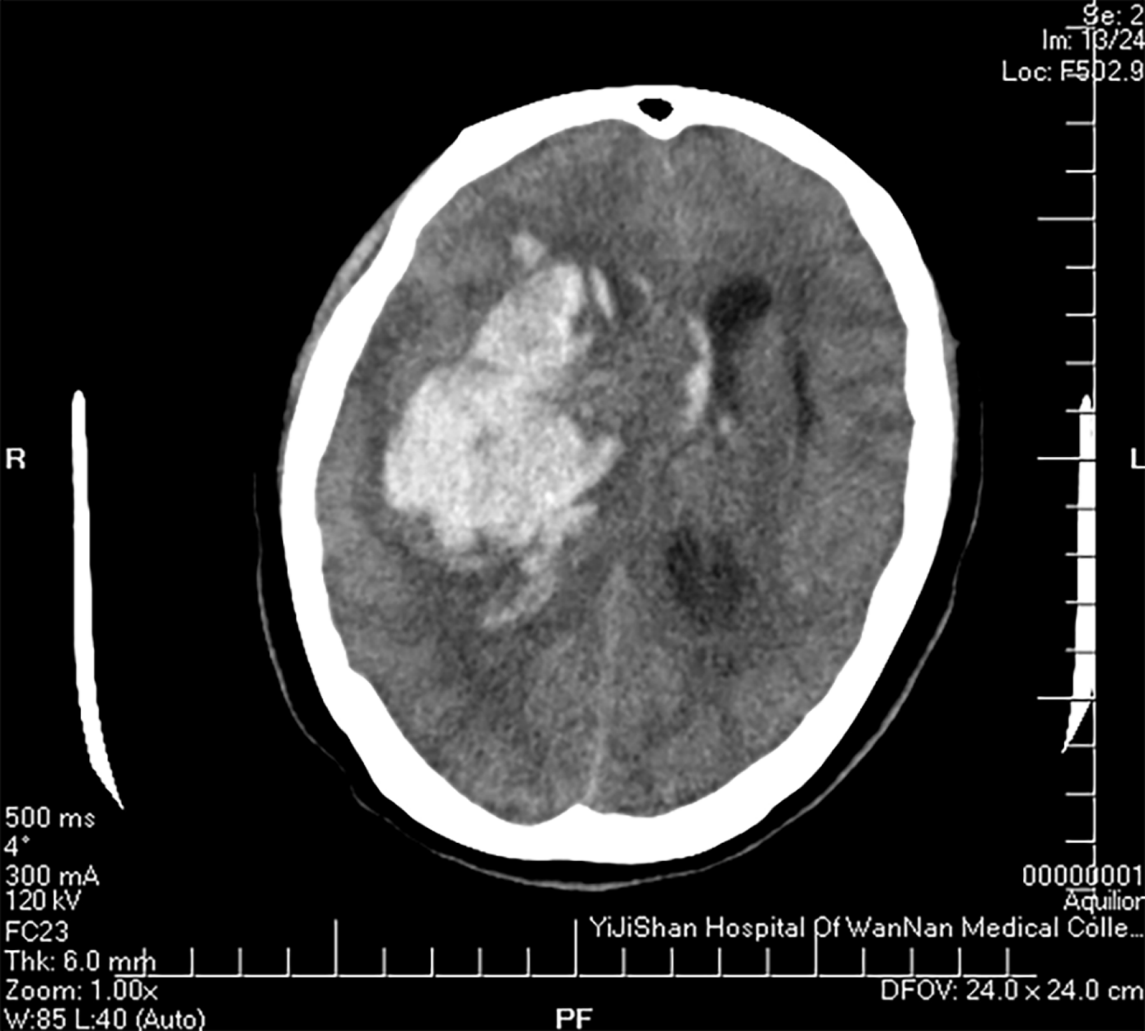
Cranial computed tomography scan showing right basal ganglia region cerebral hemorrhage

A 58-year-old woman was admitted to our hospital, because of sudden unconsciousness 1 h earlier. The patient had hypertension, and past medical history didn’t mention diabetes or heart disease. On admission, she was in the state of coma and Glasgow Coma Scale score was only 3. The prompt cranial computed tomography (CT) scan revealed right basal ganglia region cerebral hemorrhage (Fig. 1). According to the neurosurgeon, no surgical treatment was indicated. The patient was subsequently transferred to the intensive care unit with ventilator assisted respiration.

**Fig. 2:**
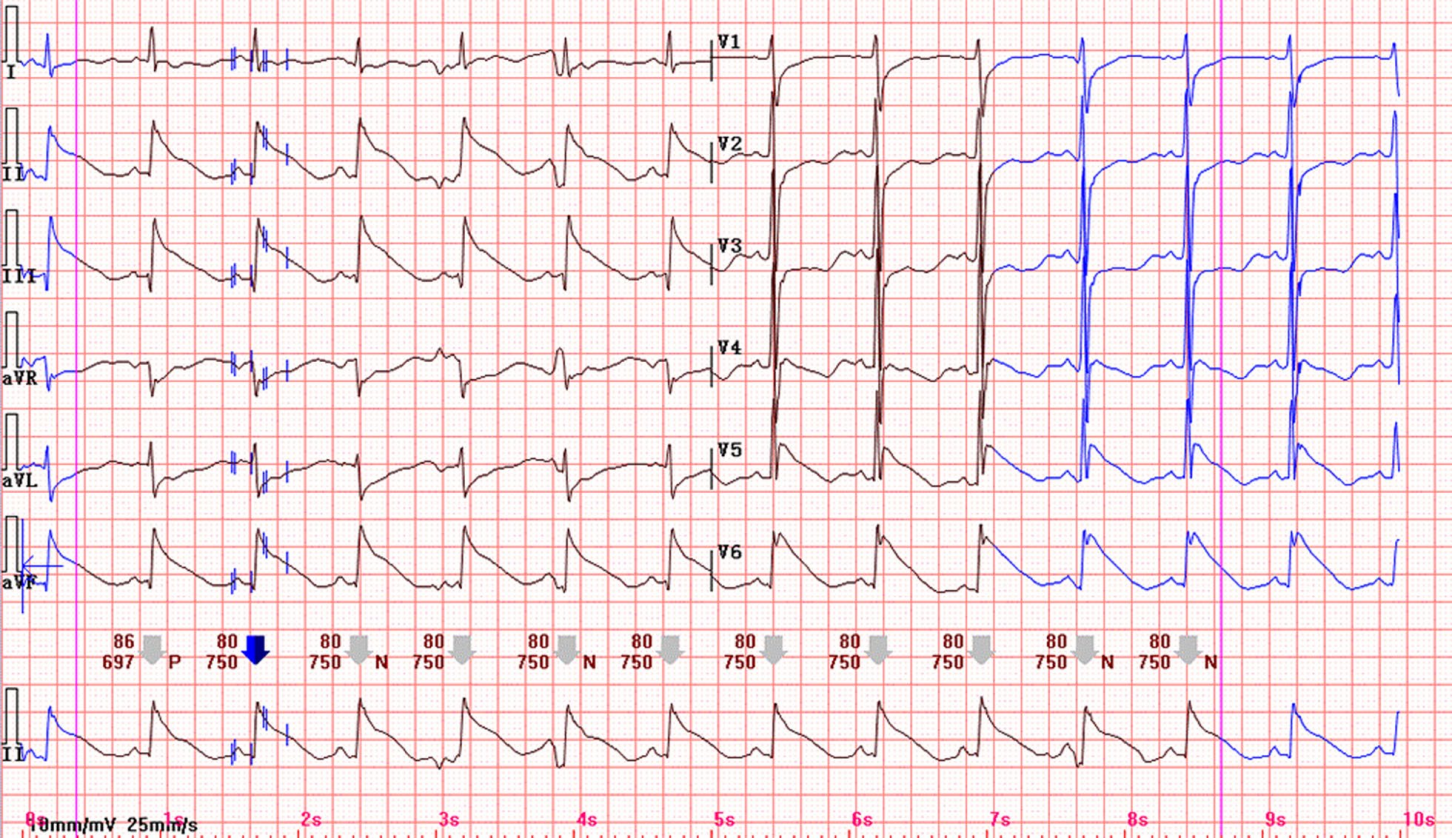
ECG showing J wave-like ST-segment elevation in leads II, III, aVF, V5, V6 and prominent J waves in leads V5, V6

**Fig. 3:**
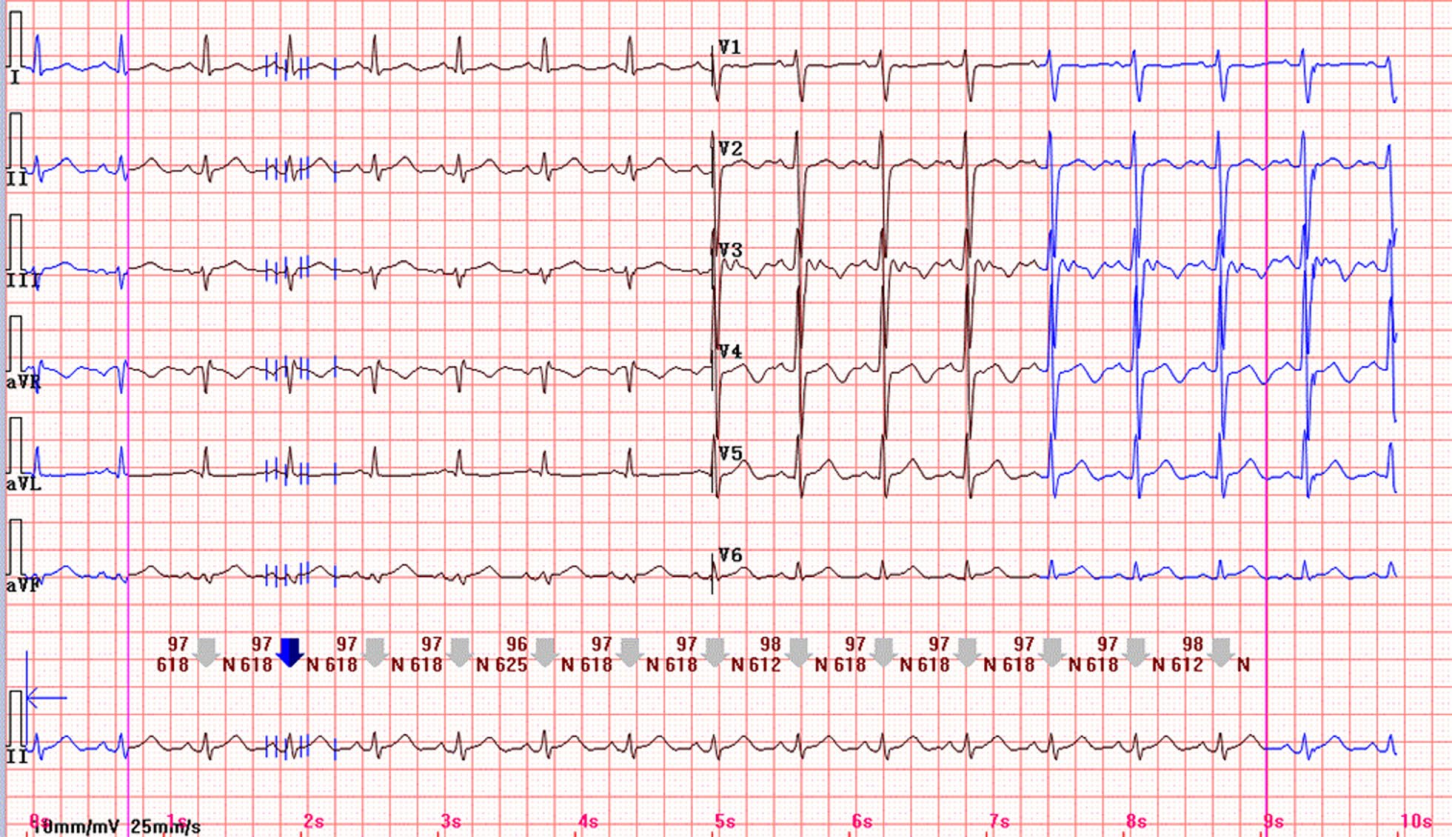
ECG showing the resolution of ST-segment elevation and J wave

The initial ECG on the day of admission showed sinus rhythm with nonspecific ST-T change. Repeated ECG on the same hospital day revealed frequent premature ventricular contractions with couplets and salves. A bedside echocardiogram demonstrated that the left ventricular systolic function was normal with an ejection fraction of 62%. Cardioselective enzymes measured at admission showed a cardiac troponin I (cTnI) of 3.44 ng/ml (reference range, 0–0.03 ng/ml), a creatine kinase (CK) of 185 U/L (reference range, 26–140 U/L) and a creatine kinase isoenzyme MB (CK-MB) of 31U/L (reference range, 0–25 U/L). On the second hospital day, the ECG exhibited J wave-like ST-segment elevation in the inferior lead (II, III and aVF) and anterolateral lead (V5, V6). Prominent J waves could be noted in leads V5 and V6 (Fig. 2). Subsequent enzyme studies showed a peak CK of 206 U/L and cTnI of 5.49 ng/ml. There was no apparent regional wall motion abnormality according to the repeated echocardiography. Coronary artery angiography was not performed since the poor general condition. The patient was treated with Isosorbide dinitrate and lipid regulators. Surprisingly, two hours later, a follow-up ECG showed that J wave-like ST-segment elevation had resolved spontaneously (Fig. 3). After two days, cTnI gradually decreased to 0.91 ng/ml. However, the patient died because of herniation of brain and central respiratory failure on the 10th day of admission.

## Discussion

Previous studies have demonstrated that cerebrovascular events are often accompanied by transient myocardial damage that may manifest as ST-segment elevation on the ECG and coinstantaneous release of cardiac troponin [5]. The J wave, described as 1 mm positive deflection at the QRS-ST junction, can be occasionally observed in patients with ICH. A prominent J wave merging into a steep downsloping ST segment without an initial upsloping phase is termed the J wave-like ST-segment elevation [6]. Several studies have suggested that some cases of acute myocardial infarction (AMI) are mechanistically linked to this distinctive ST-segment elevation pattern [7]. However, to the best of our knowledge, the J wave-like ST-segment elevation on the ECG has been rarely reported to occur in patients with ICH.

As for our case, although the increased myocardial enzyme profile and cardiac troponin implied myocardial lesion, the manifestation of the transient J wave-like ST-segment elevation without an appearance of pathological Q wave on the ECG may suggest ICH-related ST-segment elevation rather than AMI. The surge of circulating catecholamine concentration and neurological stress, could be possible mechanisms responsible for such ECG changes in patients with ICH [8]. Several lines of evidence have demonstrated increased catecholamine could lead to the spasm of coronary arteries and damage myocytes directly, causing ECG changes and associated myocardial lesion [9, 10]. Acute ischemia secondary to coronary spasm may cause a reduction of Na+ or Ca++ and an augmentation of K+ currents, leading to an increased net repolarizing current in the epicardium rather than the endocardium, giving rise to a transmural voltage gradient. As a result, ST-segment elevation with an augmentative J wave will be manifested on the ECG [11]. Also, the appearance of J wave-like ST-segment elevation in this ICH patient might be attributed to catecholamine toxicity, which could induce a mismatch leading to loss of the Ito-mediated action potential dome between endocardium and epicardium [10]. It is noteworthy that the electrical heterogeneity may give rise to the phase 2 reentry and resultant ventricular arrhythmias. In general, it is catecholamine-induced coronary artery spasm and direct cardiac myocytes toxicity that possibly mediate J wave-like ST-segment elevation on the ECG of ICH patients.

As a part of ICH, the concentration of plasma catecholamine were increased at the very onset. Following the incident, the concentration of epinephring and norepinephrine decreased gradually according to the study by Espiner et al. [12]. Subsequently, coronary perfusion would be re-established. This could explain why ECG changes along with myocardial lesion in our case were resolved spontaneously. Additionally, the above-mentioned situation should be distinguished from the Tako-tsubo syndrome that often characterizes with a reversible left ventricular wall dyskinesia at the apex following emotional or physical stress. Since the absence of left ventricular apical dyskinesia in our patient, Tako-tsubo syndrome could be ruled out.

## Conclusions

Although the presence of J wave-like ST-segment elevation is generally regarded as highly specific manifestation for primary ischemic heart diseases, it can also happen in patients with ICH. We believe that the follow-up ECGs can be used in conjunction with repeated myocardial enzyme analysis and echocardiography to diffferentiate ICH-ralated J wave-like ST-segment elevation from AMI, thus avoiding unnecessary cardiac catheterization.

## Data Availability

All relevant data supporting the conclusions of this article are included within the article.

## Abbreviations

ICH: Intracerebral hemorrhage
ECG: Electrocardiography
AMI: Acute myocardial infarction
CT: Computed tomography
cTnI: Cardiac troponin I
CK: Creatine kinase
CK-MB: Creatine kinase isoenzyme MB
